# Alanylated lipoteichoic acid primer in *Bacillus subtilis*

**DOI:** 10.12688/f1000research.8007.2

**Published:** 2016-04-11

**Authors:** Yu Luo

**Affiliations:** 1 Department of Biochemistry, University of Saskatchewan, Saskatoon, Canada

**Keywords:** Host immune response, Gram-positive, Lipoteichoic acid, D-alanylation, Glycolipid D-alanyl-phosphatidylglycerol, Surface charge, Lipoteichoic acid primer, Mass spectrometry, Lipidomics

## Abstract

Lipoteichoic acid is a major lipid-anchored polymer in Gram-positive bacteria such as *Bacillus subtilis*. This polymer typically consists of repeating phosphate-containing units and therefore has a predominant negative charge. The repeating units are attached to a glycolipid anchor which has a diacylglycerol (DAG) moiety attached to a dihexopyranose head group. D-alanylation is known as the major modification of type I and type IV lipoteichoic acids, which partially neutralizes the polymer and plays important roles in bacterial survival and resistance to the host immune system. The biosynthesis pathways of the glycolipid anchor and lipoteichoic acid have been fully characterized. However, the exact mechanism of D-alanyl transfer from the cytosol to cell surface lipoteichoic acid remains unclear. Here I report the use of mass spectrometry in the identification of possible intermediate species in the biosynthesis and D-alanylation of lipoteichoic acid: the glycolipid anchor, nascent lipoteichoic acid primer with one phosphoglycerol unit, as well as mono- and di-alanylated forms of the lipoteichoic acid primer. Monitoring these species as well as the recently reported D-alanyl-phosphatidyl glycerol should aid in shedding light on the mechanism of the D-alanylation pathway of lipoteichoic acid.

## Introduction

Phospholipids are the dominant cell membrane component in most bacteria ^1^ which render bacterial cell surface negatively charged. This feature makes bacterial membrane the easy target of host immune molecules such as cationic antibiotic peptides ^2– 4^. Bacteria have been known to constantly modulate membrane components ^1, 5^. There are at least three pathways which may contribute to surface charge modulation: biosynthesis of phosphatidylethanolamine (PE), L-lysyl-phosphatidylglycerol (lysyl-PG), and D-alanylation of lipo- and wall-teichoic acids. In comparison with Gram-negative bacteria, Gram-positive bacteria typically have noticeably less PE ^1^, but have an abundance of lysyl-PG or other aminoacylated PGs which most Gram-negative bacteria lack ^5, 6^. Besides, lipo- and wall-teichoic acids are only found in Gram-positive bacteria.

**Figure 1.**
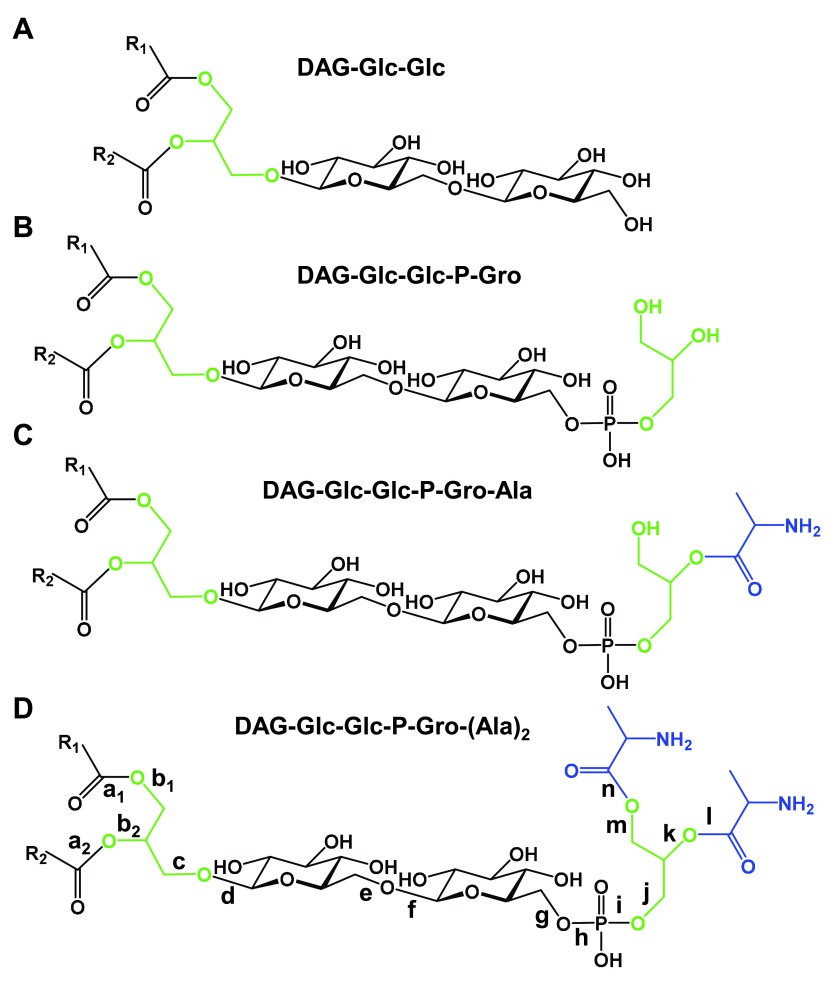
Molecular structures. Scissile bonds are labeled alphabetically in **D**. DAG – diacylglycerol; P – phosphate; Gro – glycerol; Glc – glucose; Ala – alanine. **A**. The glycolipid anchor of lipoteichoic acid: DAG-Glc-Glc. **B**. The lipoteichoic acid primer: DAG-Glc-Glc-P-Gro. **C**. Mono-alanylated lipoteichoic acid primer: DAG-Glc-Glc-P-Gro-Ala. **D**. Bis-alanylated lipoteichoic acid primer: DAG-Glc-Glc-P-Gro-(Ala) _2_.

Gram-positive bacteria lack the outer membrane as well as phosphate-rich lipid A and lipopolysaccharide found in Gram-negative bacteria. Instead they have a diverse category of polymeric teichoic acids made of phosphate-containing repeating units. Peptidoglycan-attached wall-teichoic acids and glycolipid-anchored lipoteichoic acids were discovered six decades ago ^7^. There are five types of lipoteichoic acids ^10^ and four types of wall teichoic acids ^11^. The biosynthesis pathways of the two types of teichoic acids have been characterized ^8– 11^. Glycerol or ribitol residues in the repeating units of type I and type IV lipoteichoic acids ^10^ as well as type I wall teichoic acids ^11^ are known to undergo D-alanine esterification ^7, 9, 12^, which is known to be carried out by the four *dlt* operon-coded proteins DltABCD ^13^. This surface charge modulation has been observed to significantly affect the antigenicity of the bacteria ^2^. In the cytosol, DltA (~500 amino acid residues) catalyzes with the consumption of ATP first the adenylylation of D-alanine and then the thioester formation with D-alanyl-carrier protein DltC (~80 amino acids) ^13– 15^. Crystal structures of DltA ^16, 17^ have proven that DltA is homologous to adenylation domains (also called AMP-forming domains) found in modular nonribosomal peptide synthetases ^18^ as well as fatty acyl-coenzyme A synthetases ^19^ and firefly luciferases ^20^. The functionally uncharacterized DltB (~400 amino acid residues) is predicted to be an integral membrane protein with multiple putative transmembrane helices with a low level of similarity to a putative group of membrane-bound O-acyltransferases ^21^. DltD (~400 amino acid residues), with a single putative N-terminal transmembrane helix and a large globular domain, has been reported to bind DltC and possibly catalyzes the final D-alanyl transfer from DltC to teichoic acid ^22^. We have recently characterized the presence of D- but not L-alanine in lipid lysate from *Bacillus subtilis,* implying the presence of D-alanyl-PG in the bacterial membrane ^23^. Observation of other D-alanylated species in the bacterial membrane would help sketching a transfer route for the D-alanyl group from inside the cytosol to teichoic acids on the outer surface. Here I report profiling of *B. subtilis* lipids and identification of mono- and di-alanylated derivatives of nascent lipoteichoic acid primer with a single phosphoglycerol unit attached to the glycolipid anchor (chemical structures shown in Figure 1).

## Materials and methods

*Bacterial strain and cell culture.* The BL21 (DE3) strain of *E. coli* (Novagen) and *B. subtilis* strain 168 (Bacillus Genetic Stock Center) were first plated from freezer stock onto LB-agar media. A single colony was transferred into 100 ml of LB media. After incubation overnight at 37°C and 220 rpm in an environmental shaker, it was transferred to 1 liter of LB media. When the cell density reached ~1.0 at 600 nm, 200 ml cell culture supplemented with 2.0 ml of 1.0 M NaAc buffer at pH 4.6 was centrifuged at 5,500 rpm in a Beckman JLA-8.1 rotor for 16 minutes at 4°C. The wet cell pellet was used for lipid extraction.

*Lipid extraction.* HPLC-grade organic solvents (Fisher Scientific) and distilled and deionized water were used throughout the experiment. The lipid extraction procedure was following that of Bligh and Dyer ^24^. Briefly, the wet cell pellet was re-suspended in a glass tube in 0.5 ml ice-chilled water and 2.0 ml of ice-chilled methanol. Then 1.0 ml of cold chloroform was added. The suspension was vortexed for 3 seconds every 5 minutes and incubated on ice for a duration of 10 minutes. After that, 2.0 ml cold chloroform was added followed by 1.5 ml of cold water. The tube was vortexed for 3 seconds and placed on a rocking platform at a room temperature of 21°C for 3 minutes. Phase separation was assisted by centrifugation at 1,300 rpm for 5 minutes with a Beckman Allegro X-22R centrifuge. The heavier chloroform-rich phase was transferred by a glass syringe to a second glass centrifuge tube. Another 2.0 ml cold chloroform was added to the first tube and vortexed for 3 seconds. Then the first tube was put back on the rocking platform at room temperature for 10 minutes. Centrifugation at 1,300 rpm for 5 minutes and transfer of the heavier chloroform-rich phase to the second glass tube followed. The combined chloroform-rich phase was mixed with 0.5 ml 0.5 M NaCl, vortexed for 3 seconds and gentle shaking by hand for 1 minute. After centrifugation at 1,300 rpm for 5 minutes, the chloroform-rich phase, 4.0–4.5 ml in volume, was collected in a third glass tube for storage at -80°C. Typically, the total lipid concentration was estimated as 0.5 mg/ml.

*Lipid profiling by mass spectroscopy.* The lipid samples were diluted by adding 2-fold volume of methanol to a concentration of ~0.15 mg/ml (or 150 ppm) for direct infusion at a rate of 0.6 ml/hour to a SCIEX 4000 QTRAP mass spectrometer. Electrospray ionization was achieved at a temperature of 500°C and a pressure of 20 psi for curtain gas as well as ion source gas 1 and 2. The collision energy in the ion trap was tested between 30 and 100 electronvolts for most efficient detection of target substructures in the lipids. The SCIEX Analyst 1.6 software was used to acquire and export averaged mass spectra with the 4000 QTRAP system. Agilent MassHunter B.06.00 was used to process mass spectra with an Agilent Q-TOF 6500 system. MS spectra in the figures were also analyzed with Mass++ 2.7.4 software ^25^ and presented with Microsoft Excel.

*Tandem mass spectroscopy.* The targeted MS/MS spectra were first acquired using the SCIEX 4000 QTRAP system with multiple collision energy settings between 50 and 90 electronvolts. High-accuracy MS/MS spectra were acquired using the Agilent Q-TOF 6550 system with collision energy ranging from 30 to 80 electronvolts. Direct infusion was also employed but at a faster flow rate of 2.0 ml/hour for the Q-TOF 6550 system.

## Results

Raw data for ‘Alanylated lipoteichoic acid primer in *Bacillus subtilis*’, Luo 2016 README.txt contains a description of the files. Click here for additional data file. Copyright: © 2016 Luo Y 2016 Data associated with the article are available under the terms of the Creative Commons Zero "No rights reserved" data waiver (CC0 1.0 Public domain dedication).

*Polar lipid extraction on ice produced more species in the sample* - Ice-chilled solvents instead of room-temperature ones were used during the well-established polar lipid extraction procedure devised by Bligh and Dyer ^24^. The new lipid preparations did not show marked differences on thin-layer chromatograms. However, their mass spectra showed noticeable difference with the cold extraction producing more species than room-temperature extraction. The alanylated derivatives of lipoteichoic acid primer were not observed in lipids extracted at room temperature.

**Figure 2.**
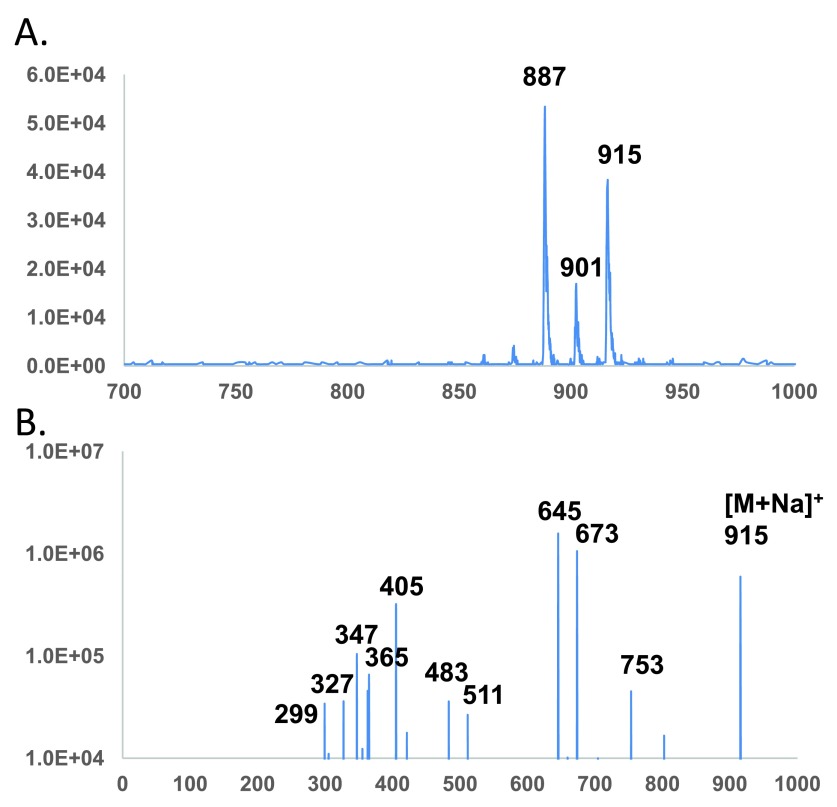
Lipid profiling and tandem mass spectra of sodiated DAG-Glc-Glc. Horizontal axis denotes m/z values. Vertical axis denotes ion counts. DAG – diacylglycerol; Glc – glucose. **A**. Precursor scan for 347 amu sodiated diglucose dehydrate. **B**. MS/MS spectrum of sodiated (32:0) DAG-Glc-Glc (915 amu).

*Profiling and tandem mass spectroscopy of polar lipids with dihexose head group* - The sodiated form of the lipid anchor of lipoteichoic acid in *B. subtilis* has been identified by mass spectrometry previously ^26^. Several mass spectrometric scans with the 4000 QTRAP system in search for the lipid anchor were experimented. The anchor has a common structure of DAG-dihexose, with the hexose being either glucose or galactose depending on the identity of the microbial organism ^27^. The unbranched and typically glycerolphosphate polymer is attached to C-6 of the non-reducing hexopyranosyl end of the glycolipid anchor by a phosphodiester bond ^27^. In *B. subtilis*, the head group is diglucose ^27^ ( Figure 1A). The sodiated dehydrated diglucose (342 – 18 + 23 = 347 amu) at a collision energy of +80 electronvolts revealed the two most intense peaks (887 and 915 amu) matching expected sizes of the lipid anchor with the two dominant fatty acyl compositions of (30:0) and (32:0), respectively ^26^ ( Figure 2A). Tandem mass spectra of the most abundant 915 amu species was then acquired with the SCIEX 4000 QTRAP system ( Figure 2B) and the Agilent Q-TOF 6550 system ( Table 1). The QTRAP system produced less noisy spectra which are shown in Figure 2– Figure 4. The m/z values obtained from the Q-TOF system were more accurate and are listed in Table 1– Table 4. The observed molecular mass 915.601 closely matched the calculated value of 915.603 for (32:0) [DAG-Glc-Glc + Na] ^+^. All the observed fragment ions also had their m/z values within 0.002 amu of calculated monoisotopic masses. The molecular ion dissociated to form two most abundant fragments at 645 and 673 amu, corresponding to neutral loss of (17:0) fatty acid (270 amu) and (15:0) fatty acid (242 amu), respectively. The two fatty acids have been known as the dominant ones in *B. subtilis* lipids ^23^. The 753 amu fragment ion corresponding to a neutral loss of a dehydrated glucose residue (162 amu) was less abundant than the twin peaks. Another set of twin peaks at 483 and 511 amu corresponded to neutral losses of the terminal glucose residue (162 amu) and either of the two fatty acids (270 and 242 amu respectively). The peak at 405 amu corresponded to the sodiated diglucose head group covalently linked with the didehydroxyl residue of glycerol CH2=CH-CH2-Glc-Glc ( Table 1). The twin peaks at 365 and 347 amu corresponded to the sodiated diglucose head group and its dehydrated form, respectively. It is worth noting that the signature [DAG – OH] ^+^ ion (551 amu) for glycerolphospholipids was missing. However, the two [MAG – OH] ^+^ ions at 299 and 327 amu were observed at lower intensity. Even though the 405 amu ion was more intense than the 347 ion, lipid profiling by searching for precursors of the 405 amu cation was inferior to the precursor scan for the 347 amu cation.

**Table 1.** Accurate masses of fragments from (32:0) [DAG-Glc-Glc + Na] ^+^.

Observed mass	Calculated mass	Cleavage	Description
299.2568	299.2588	a _1_ & c	(15:0) MAG - OH
327.2885	327.2901	a _2_ & c	(17:0) MAG - OH
347.0944	347.0955	d	Glc-Glc – H _2_O + Na ^+^
365.1056	365.1060	c	Glc-Glc + Na ^+^
405.1360	405.1374	b _1_ & b _2_	CH _2_=CH-CH _2_-Glc-Glc + Na ^+^
483.2907	483.2936	b _1_ & f	(15:0) MAG-Glc – H _2_O + Na ^+^
511.3233	511.3249	b _2_ & f	(17:0) MAG-Glc – H _2_O + Na ^+^
645.3448	645.3464	b _1_	(15:0) MAG-Glc-Glc – H _2_O + Na ^+^
673.3760	673.3777	b _2_	(17:0) MAG-Glc-Glc – H _2_O + Na ^+^
753.5487	753.5496	f	(32:0) DAG-Glc + Na ^+^
915.6005	915.6025	[M + Na] ^+^	(32:0) DAG-Glc-Glc + Na ^+^

Note: The alphabetically labeled scissile bonds are shown in Figure 1D. P – phosphate; Gro – glycerol; MAG – monoacylglycerol; DAG – diacylglycerol; Glc – glucose. There are equivalent choices such as between a _1_ and a _2_, as well as between b _1_ and b _2_.

**Figure 3.**
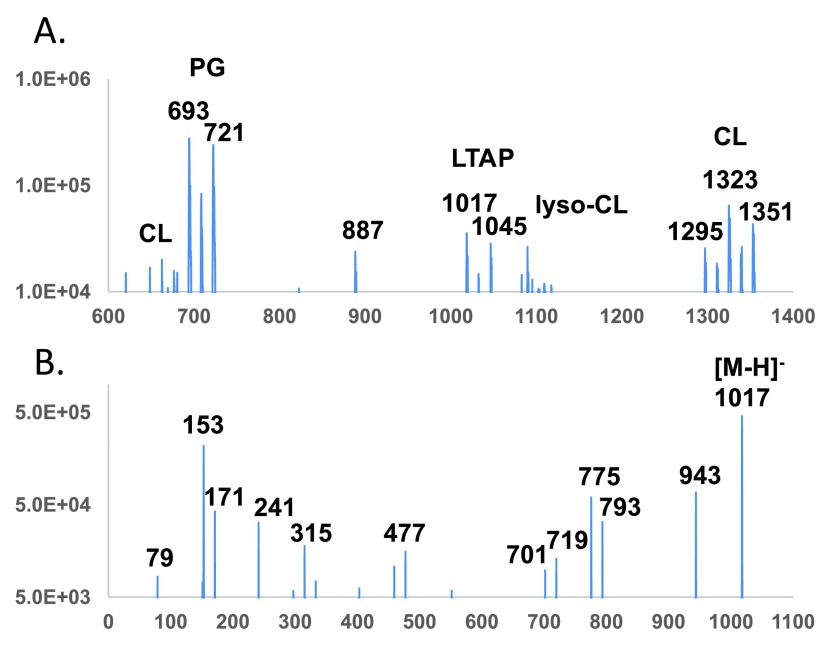
Lipid profiling and tandem mass spectra of deprotonated DAG-Glc-Glc-P-Gro lipoteichoic acid primer. Horizontal axis denotes m/z values. Vertical axis denotes ion counts. DAG – diacylglycerol; PG – phosphatidylglycerol; P – phosphate; Gro – glycerol; Glc – glucose; LTAP – lipoteichoic acid primer; CL – cardiolipin. **A**. Precursor scan for 153 amu cyclo-glycerolphosphate anion. **B**. MS/MS spectrum of lipoteichoic acid primer (30:0) DAG-Glc-Glc-P-Gro (1017 amu).

**Figure 4.**
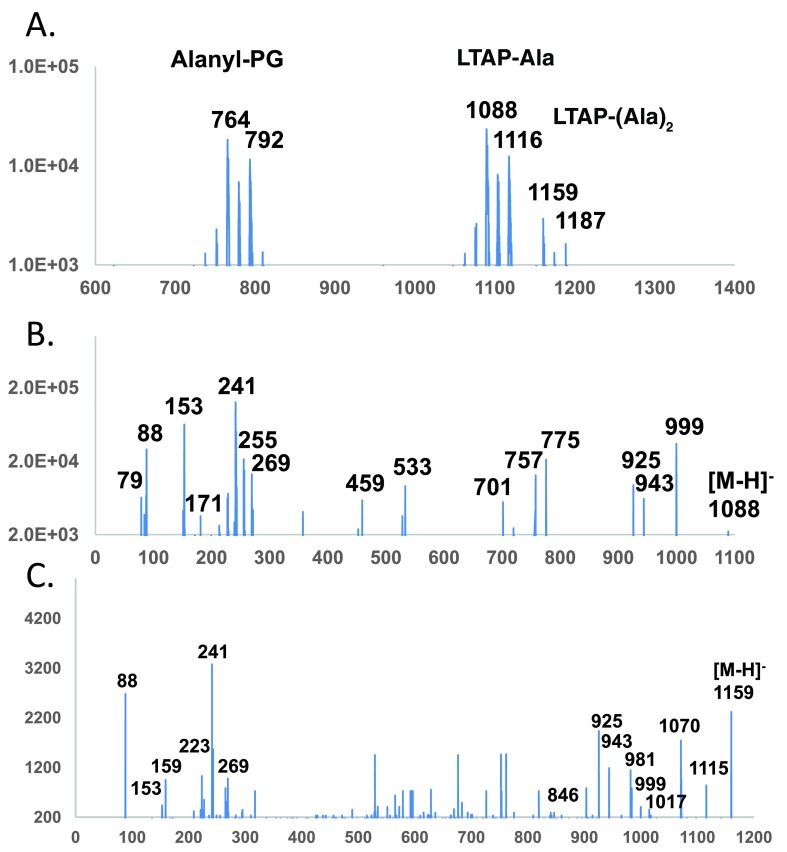
Lipid profiling and tandem mass spectra of mono- and dialanyl-derivatives of lipoteichoic acid primer. Horizontal axis denotes m/z values. Vertical axis denotes ion counts. DAG – diacylglycerol; PG – phosphatidylglycerol; P – phosphate; Gro – glycerol; Glc – glucose; Ala – alanine; LTAP – lipoteichoic acid primer. **A**. Precursor scan for 88 amu [Ala-H] ^-^. **B**. MS/MS spectrum of mono-alanylated (30:0) lipoteichoic acid primer DAG-Glc-Glc-P-Gro-Ala (1088 amu). **C**. MS/MS spectrum of dialanylated (30:0) lipoteichoic acid primer DAG-Glc-Glc-P-Gro-(Ala)2 (1159 amu).

**Table 2.** Accurate masses of fragments from (30:0) DAG-Glc _2_-P-Gro lipoteichoic acid primer.

Observed mass	Calculated mass	Cleavage	Description
78.9589	78.9585	g & i	P – H _3_O
152.9959	152.9953	h	P-Gro – H _3_O
171.0062	171.0059	g	P-Gro – H
241.2171	241.2169	b _1_ or b _2_	(15:0) FA – H
315.0463	315.0481	f	Glc-P-Gro – H _3_O
477.1002	477.1010	d	Glc _2_-P-Gro – H _3_O
701.3100	701.3051	b _1_ & j	(15:0) MAG-Glc _2_-P – H _3_O
719.3195	719.3257	a _1_ & j	(15:0) MAG-Glc _2_-P – H
775.3502	775.3519	b _1_	(15:0) lyso-form – H _3_O
793.3617	793.3625	a _1_	(15:0) lyso-form – H
943.5303	943.5399	j	DAG-Glc _2_-P – H
1017.5769	1017.5770	[M-H] ^-^	(30:0) LTAP - H

Note: The alphabetically labeled scissile bonds are shown in Figure 1D. FA – fatty acid; P – phosphate; Gro – glycerol; MAG – monoacylglycerol; DAG – diacylglycerol; Glc – glucose; LTAP – lipoteichoic acid primer DAG-Glc _2_-P-Gro. Cleavage at a _1_ and a _2_, as well as at b _1_ and b _2_ produces fragments of identical sizes.

**Table 3.** Accurate masses of fragments from (30:0) DAG-Glc _2_-P-Gro-Ala.

Observed mass	Calculated mass	Cleavage	Description
78.9587	78.9585	g & i	P – H _3_O
88.0405	88.0399	k	Ala – H
152.9959	152.9953	g & k	P-Gro – H _3_O
171.0062	171.0059	g & l	P-Gro – H
224.0316	224.0324	h	P-Gro-Ala – H _3_O
241.2170	241.2169	b _1_ or b _2_	(15:0) FA – H
459.0897	459.0904	d & k	Glc _2_-P-Gro – H _2_O – H _3_O
533.1236	533.1272	b _1_ & b _2_ & l	dilyso-LTAP – H _2_O – H _3_O
(701.4)	701.3051	b _1_ & j	(15:0) MAG-Glc _2_-P – H _3_O
719.3294	719.3257	a _1_ & j	(15:0) MAG-Glc _2_-P – H
757.3343	757.3414	b _1_ & k	(15:0) lyso-LTAP – H _2_O – H _3_O
775.3481	775.3519	b _1_ & l	(15:0) lyso-LTAP – H _3_O
925.5254	925.5293	i	(30:0) DAG-Glc _2_-P – H _3_O
943.5386	943.5399	j	(30:0) DAG-Glc _2_-P – H
999.5662	999.5661	k	(30:0) LTAP – H _3_O
1088.6146	1088.6140	[M-H] ^-^	(30:0) LTAP-Ala – H

Note: FA – fatty acid; P – phosphate; Gro – glycerol; MAG – monoacylglycerol; DAG – diacylglycerol; Glc – glucose; Ala – alanine; LTAP - LTA primer DAG-Glc _2_-P-Gro. Values in parentheses were observed only with the low-accuracy 4000 QTRAP system.

**Table 4.** Accurate masses of fragments from (30:0) DAG-Glc _2_-P-Gro-(Ala) _2_.

Observed mass	Calculated mass	Cleavage	Description
88.0401	88.0399	k or m	Ala – H
152.9946	152.9953	g & k & n	P-Gro – H _3_O
159.0762	159.0770	Figure 5A	Ala-Ala – H
223.1704	223.1698		(14:2) FA – H
241.2166	241.2169	b _1_ or b _2_	(15:0) FA – H
(846.4)	846.3891	b _1_ & l	(15:0) MAG-Glc _2_-P-Gro-Ala – H _3_O
(925.5)	925.5293	i	(30:0) DAG-Glc _2_-P – H _3_O
(943.5)	943.5399	j	(30:0) DAG-Glc _2_-P – H
(981.6)	981.5550	k & m	(30:0) LTAP – H _5_O _2_
(999.5)	999.5661	k & n	(30:0) LTAP – H _3_O
1017.5774	1017.5770	l & n	(30:0) LTAP – H
1070.6092	1070.6030	k or m	(30:0) LTAP-Ala – H _3_O
(1115.7)	1115.6610	Figure 5B	(30:0) LTAP-Ala _2_ – CO _2_ – H
1159.6527	1159.6510	[M-H] ^-^	(30:0) LTAP-Ala _2_ – H

Note: The alphabetically labeled scissile bonds are shown in Figure 1. FA – fatty acid; P – phosphate; Gro – glycerol; MAG – monoacylglycerol; DAG – diacylglycerol; Glc – glucose; Ala – alanine; LTAP – LTA primer DAG-Glc _2_-P-Gro. Values in parentheses were observed only with the low-accuracy 4000 QTRAP system.

*Profiling and tandem mass spectrometry of lipids with phosphoglycerol terminus* – The phosphoglycerol head group has a molecular mass of 172 and produced a cyclic, equivalent to dehydrated, residual anion at 153 amu. The 153 amu fragment peak is most intense for phospholipids with a terminal phosphoglycerol, and weak for phospholipids - such as cardiolipin (CL) and aminoacylated PGs - with such an embedded group. This scan between 400 and 1700 amu at a collision energy of -95 electronvolts was most effective in hitting larger precursor ions (part of the mass range is shown in Figure 3A). The spectrum revealed a cluster of cardiolipin (CL) double anions in the 650–680 amu range and a more intense cluster centered around two major anions at 693 and 721 amu, corresponding to the dominant lipids of (30:0) and (32:0) PGs, respectively. There was an 887 amu unknown species as well as mostly dehydrated lyso-cardiolipins (lyso-CL) close to 1100 amu and cardiolipins close to 1300 amu. There were no noticeable hits below 600 amu or between 1400 and 1700 amu. Besides, the 1017 amu and 1045 amu anions matched expected masses of lipoteichoic acid primer ( Figure 1B) with dominant fatty acyl compositions of (30:0) and (32:0), respectively.

The 1017 amu anion had two identical (15:0) fatty acyl chains and therefore made assignment of fragments less difficult. The MS/MS spectra of the 1017 ion acquired with the QTRAP system at a collision energy of -90 electronvolts is shown in Figure 3B, and m/z values of fragments are listed in Table 2. In addition to the 79 amu phosphate residue, the pair of 153 amu and 171 amu ions which corresponded to glycerolphosphate residue, the dominant fatty acid ion at 241 amu matched the expected (15:0) composition. Fragmentation at the two glycosyl bonds likely produced the 315 amu and 477 amu ions. At the other end of the spectrum, the 943 amu ion was likely due to the neutral loss of cycloglycerol (74 amu). A further loss of (15:0) fatty acid (242 amu) or ketene (224 amu) likely produced the pair of 701 and 719 amu ions, respectively. Another pair at 775 and 793 amu were produced similarly but from the molecular ion. The 1017 amu molecular ion matched structural characteristics of a lipoteichoic acid primer with a single glycerolphosphate unit attached to the lipid anchor of diglucosyldiacyglycerol.

*Profiling of lipids with ester-linked alanine* – In negative mode, ester-linked fatty acids are known to form intense fragment [FA-H] ^-^ ions. This is also true for ester-linked amino acids ^23^. A precursor scan between 400 and 1700 amu at an optimized collision energy of -95 electronvolts for 88 amu [Alanine-H] ^-^ (part of the mass range is shown in Figure 4A) revealed as expected a cluster of alanyl-PGs with two dominant peaks at 764 and 792 amu corresponding to (30:0) and (32:0) alanyl-PG, respectively. The precursor scan also revealed two adjacent clusters of alanylated lipids separated by 71 amu which corresponded to the molecular mass of a dehydrated alanine. The first cluster with dominant 1088 and 1116 amu anions matched expected m/z values of mono-alanylated lipoteichoic acid primers ( Figure 1C), while the second cluster centered around the 1159 and 1187 amu anions matched those of di-alanylated lipoteichoic acid primers ( Figure 1D). There were no noticeable hits below 700 amu or between 1200 and 1700 amu.

The overall mass of the 1088 amu anion matched that of (30:0) alanyl-lipoteichoic acid primer. The MS/MS spectra of the 1088 ion acquired with the QTRAP system at a collision energy of -90 electronvolts is shown in Figure 4B, and m/z values of fragments are listed in Table 3. The 79, 153 and 171 amu ions corresponded to the putative glycerolphosphate backbone of this lipid. The dominant fatty acid ion at 241 amu matched the expected (15:0) composition and its putative ester linkage to the lipid. The 88 amu ion implied a terminal ester-linked alanine. The 224 amu ion corresponded to the dehydrated or cyclic form of the putative head group of alanylated glycerolphosphate. At the other end of the spectrum, the 999 amu ion was likely due to the neutral loss of alanine (89 amu) from the 1088 molecular anion. The pair of ions at 925 and 943 amu corresponded to the neutral loss of linear (163 amu) or cyclic (145 amu) alanyl-glycerol. In the mid-section of the spectrum, the 459 amu species corresponded to neutral losses of both DAG (540 amu) and alanine (89 amu), while the 533 amu ion corresponded to neutral losses of both fatty acids (2 × 242 = 484 amu) and a dehydrated alanine (71 amu). Fragments at 757 and 775 amu corresponded to the loss of one (15:0) fatty acid or ketene (242 or 224 amu) as well as alanine (89 amu). The smaller 701 and 719 amu fragments corresponded to loss of one (15:0) fatty acid or ketene as well as cyclo-alanyl-glycerol (145 amu). Except for the absence of 701 amu anion and the low-abundance 757 amu anion in the Q-TOF-acquired spectrum, all fragments matched expected m/z values within 0.004 amu. This 1088 amu is most likely mono-alanylated lipoteichoic acid primer.

**Figure 5.**
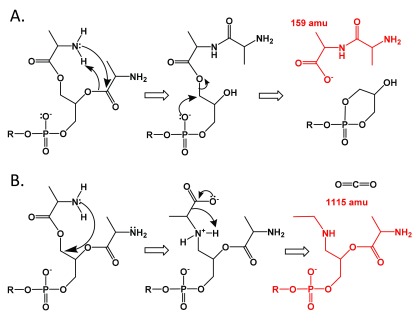
Putative two-step fragmentation reactions. The DAG-Glc-Glc part is shown as R. **A**. Rearrangement and fragmentation to produce the 159 amu dialanyl anion. **B**. Rearrangement and decarboxylation reactions which resulted in the neutral loss of the 44 amu CO _2_ and the 1115 amu fragment anion.

The m/z value of the 1159 amu anion matched that of (30:0) di-alanyl-lipoteichoic acid primer. The MS/MS spectra of the 1159 ion acquired with the QTRAP system at a collision energy of -80 electronvolts is shown in Figure 4C, and m/z values of fragments are listed in Table 4. Due to the lower abundance of the 1159 anion, its MS/MS spectrum was noisier than those of the 1017 and 1088 anions. The spectrum shared 79, 88, 153, 171 and 241 amu ions with that of the 1088 amu anion. Unexpectedly, a 159 amu species corresponding precisely to deprotonated alanylalanine dipeptide was also observed. The mid-section of the spectrum did not reveal reoccurring ions in spectra collected at collision energies 10 electronvolts apart and therefore were likely noise due to the low abundance of this molecular ion. The whole head group of dialanylated glycerolphosphate was not observed. At the high end of the spectrum, an 846 amu fragment was probably generated by neutral losses of both (15:0) fatty acid (242 amu) and a cyclo-alanine (71 amu). The 925, 943 and 999 amu ions were in common with the fragments from the 1088 amu anion of mono-alanylated lipoteichoic acid primer. A further dehydrated 981 amu ion was observed, which corresponded to neutral losses of two alanine molecules (2 × 89 = 178 amu). A larger 1017 amu ion precisely matched that of (30:0) lipoteichoic acid primer. Another even larger 1070 amu ion corresponded to the 89 amu neutral loss of one alanine from the 1159 amu molecular anion. Surprisingly, a 1115 amu ion corresponding to a neutral loss of 44 amu was observed. As shown in Figure 5A, a two-step reaction may account for the formation of alanylalanyl-LTA primer and subsequent fragmentation into the 159 amu alanylalanine anion. An alternate reaction shown in Figure 5B may rearrange the putative bis-alanyl-LTA primer to expose a terminal carboxyl group in one of the two alanine residues, which could subsequently release the 44 amu CO _2_ and produce the 1115 amu fragment anion. Due to the lack of any fragment ion corresponding to linear (313 amu) or cyclic (295 amu) bis-alanyl-glycerolphosphate, the result was not definitive on the location of two ester-linked alanine residues. Based on its similar fragmentation pattern to that of mono-alanylated LTA primer, this 1159 amu species is tentatively assigned as bis-alanyl-LTA primer ( Figure 1D).

## Discussion

Aminoacylated lipids play an apparent role in surface charge modulation of Gram-positive bacteria ^5^. The least known part of charge modulation is arguably the D-alanylation pathway of lipoteichoic acids. The Bligh and Dyer method ^24^ carried out at an icy temperature appeared to be essential for successful extraction of species that are almost certainly lipoteichoic acid primer and its mono- and di-alanylated derivatives. My lab has recently observed that lysate of *B. subtilis* lipids contained predominantly D-alanine. Lysate of lipids in this study showed the expected predominance of D-alanine over L-alanine. The lipoteichoic acid primers were possibly esterized with D-alanine. The possible existence of the putative bis-alanylated lipoteichoic acid primer indicates that these species are unlikely to be hydrolyzed fragments of lipoteichoic acids since hydrolysis could only produce mono-alanylated derivative. Hydrolysis of lipoteichoic acid should also produce detectable amount of residue with more than one phosphoglycerol units attached to the lipid anchor, which was apparently lacking in the lipid extract. It also implies that lipoteichoic acid is unlikely to be transferred as D-alanyl-glycerolphosphate unit directly from D-alanyl-PG to the growing lipoteichoic acid chain by the LtaS polymerase as that would only produce mono-alanylated derivative. The observable abundance of lipoteichoic acid primer also appear to suggest that one of the four LtaS paralogs in *B. subtilis* ^28^ may indeed act like LtaP primase in *Listeria monocytogenes* for the biosynthesis of lipoteichoic acid primer ^29^.

**Figure 6.**
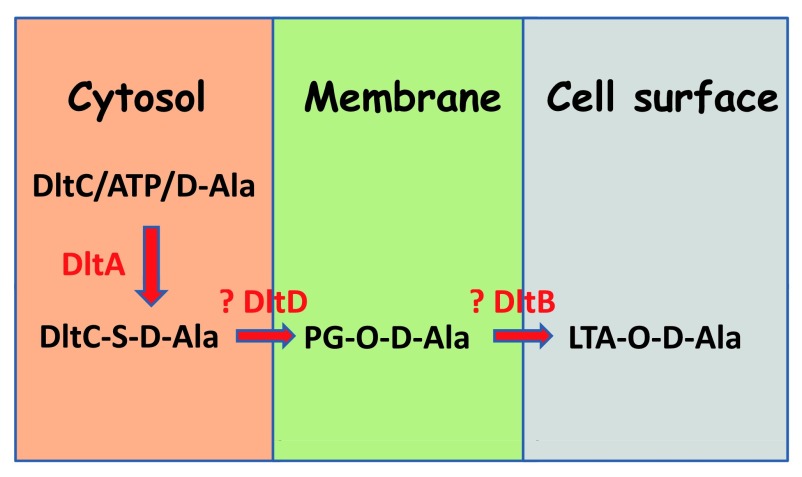
Putative D-alanylation pathway of lipoteichoic acid. DltA catalyzes the loading of thioester-linked D-alanine to the carrier protein DltC. DltC-carried D-alanyl group is further transferred to PG in the membrane by forming an ester bond. The putative enzyme for this process is DltD. PG-attached D-alanyl group is further transferred to LTA by transesterification. The putative enzyme for this latter process is DltB.

My lab has recently hypothesized that D-alanyl-PG may serve as the lipid intermediate for subsequent D-alanylation of teichoic acids. Taken together, a putative pathway is shown in Figure 6. It is known that DltA catalyzes the activation of D-alanine with the consumption of ATP and thioester formation with the D-alanyl carrier protein DltC. It is possible either DltD or DltB - with the former being more likely based on the best available evidences that DltD binds DltC and has thioesterase activity ^22^ – catalyzes the transfer of thioester-bound D-alanyl group to PG in the bacterial membrane by a thermodynamically spontaneous esterification reaction. The other one of the pair of Dlt proteins, most likely DltB, then catalyzes the transfer of D-alanyl group from the PG carrier to lipoteichoic acid by a transesterification reaction that can only reach equilibrium. This thermodynamic nature of this final transesterification reaction would enable the accumulation of a significant amount of the D-alanyl-PG intermediate, which is consistent with my lab’s recent observation that alanyl-PG is somewhat abundant in lipids extracted from *B. subtilis*. Importantly, the diglucosyl-diacylglycerol anchor, lipoteichoic acid primer, D-alanylated lipoteichoic acid primer as well as D-alanylated phosphatidylglycerol can be monitored in lipids extracted from wild-type and mutant cells of *B. subtilis* and aid in the full elucidation of the D-alanylation pathway of lipoteichoic acids.

## Data availability

The data referenced by this article are under copyright with the following copyright statement: Copyright: © 2016 Luo Y

Data associated with the article are available under the terms of the Creative Commons Zero "No rights reserved" data waiver (CC0 1.0 Public domain dedication). 

*F1000Research*: Dataset 1. Raw data for ‘Alanylated lipoteichoic acid primer in *Bacillus subtilis*’, Luo 2016. README.txt contains a description of the files, 10.5256/f1000research.8007.d113434 ^30^
