# Supplementary material for: Alanylated lipoteichoic acid primer in Bacillus subtilis
Source: F1000Res. 2016 Apr 11;5:155. Originally published 2016 Feb 10. [Version 2] doi: 10.12688/f1000research.8007.2 (PMC4837988; doi:10.12688/f1000research.8007.2)
Supplement: Peer review history

# Peer review: Referee response for version 2

**DOI:** 10.5256/f1000research.9104.r13311

**Published:** 2016-04-14

**Authors:** Christian Sohlenkamp

**Affiliations:** 1 Center for Genomic Sciences, National Autonomous University of Mexico (UNAM), Cuernavaca, Mexico

I have no further objections to the indexing of the article.

I have read this submission. I believe that I have an appropriate level of expertise to confirm that it is of an acceptable scientific standard.

# Peer review: Referee response for version 2

**DOI:** 10.5256/f1000research.9104.r13312

**Published:** 2016-04-13

**Authors:** Katarzyna Duda

**Affiliations:** 1 Junior Group of Allergobiochemistry, Research Center Borstel, Leibniz-Center for Medicine and Biosciences, Airway Research Center North (ARCN), Borstel, Germany

I am satisfied with the Author responses to my comments, and respective corrections.

I have read this submission. I believe that I have an appropriate level of expertise to confirm that it is of an acceptable scientific standard.

# Peer review: Referee response for version 1

**DOI:** 10.5256/f1000research.8616.r12358

**Published:** 2016-03-24

**Authors:** Christian Sohlenkamp

**Affiliations:** 1 Center for Genomic Sciences, National Autonomous University of Mexico (UNAM), Cuernavaca, Mexico

The present manuscript describes a mass spectrometric study of Bacillus subtilis lipids in which possible intermediates in the biosynthesis and D-alanylation of type I lipoteichoic acid are detected.

In general, the title is appropriate, and the abstract provides an adequate summary of the article. The conclusions are based on the raw data presented. My major critique is that the author actually does not show the presence of the pathway, he only detects molecular ions my MS that might be pathway intermediates. The other comments should be addressed to improve the manuscript.

Comments:

1. Abstract: I think the word “possible” should be added, “Here I report the use of mass spectrometry in the identification of possible intermediate species…”, because although that it is very probable that this is case, strictly speaking, it has not been shown.
2. Abstract: In the same sense, I think it is preferable to write “…D-alanyl-phosphatidyl glycerol should aid in…”
3. Introduction: It should be specified that the author refers to type I LTA.
4. Introduction: The first part of the introduction is very simplified, for example no lipid A is mentioned, nor the modification it can suffer to make it less anionic. I suggest rewriting and improving this paragraph.
5. Figure 1 and 5: Double bonds should look identical in the figures.
6. Strictly speaking the authors don’t show that it is D-alanine in the detected structures.
7. Results/Discussion: What does the author think is the explanation that the new species only can be detected after a cold extraction?
8. Methods: Why do the authors add sodium acetate to the growth medium before centrifugation of the culture?
9. Page 2, left column, middle paragraph: I think it should say “adenylylation” not “adenylation”, because it is an AMP-transfer.
10. Page 2, left column, middle paragraph: Instead of “The functionally uncharacterized DltB appears to be an integral membrane protein…”, it would be better to write “is predicted to be…”.
11. Page 2, left column, middle paragraph: It should say “O-acyltransferase”, not “o-acyltransferase”
12. In the legends to figures 3 and 4, it should say “acid” not “aicd”.
13. Methods/Results: Why are the electronvolts sometimes “+” and sometimes “-“
14. Tables 2 and 4: The abbreviation FA for fatty acid should be explained.
15. Page 8, left column, middle paragraph: The word “smaller” should be deleted.
16. I agree with reviewer one that P is probably a better abbreviation of the phosphate group. 

I have read this submission. I believe that I have an appropriate level of expertise to confirm that it is of an acceptable scientific standard, however I have significant reservations, as outlined above.

# Response

**Published:** 2016-03-28

**Authors:** Yu Luo

**Affiliations:** Department of Biochemistry, University of Saskatchewan, Canada

**Christian Sohlenkamp**, Center for Genomic Sciences, National Autonomous University of Mexico (UNAM), Mexico 

The present manuscript describes a mass spectrometric study of Bacillus subtilis lipids in which possible intermediates in the biosynthesis and D-alanylation of type I lipoteichoic acid are detected.

In general, the title is appropriate, and the abstract provides an adequate summary of the article. The conclusions are based on the raw data presented. My major critique is that the author actually does not show the presence of the pathway, he only detects molecular ions my MS that might be pathway intermediates. The other comments should be addressed to improve the manuscript.

**YL: I appreciate Dr. Sohlenkamp’s comments. I will incorporate changes as described below in the revised version.**

Comments:

1.      Abstract: I think the word “possible” should be added, “Here I report the use of mass spectrometry in the identification of possible intermediate species…”, because although that it is very probable that this is case, strictly speaking, it has not been shown.

**YL: I agree that its status as an intermediate has not been demonstrated so that “possible” should be added.**

2.     Abstract: In the same sense, I think it is preferable to write “…D-alanyl-phosphatidyl glycerol should aid in…”.

**YL: I agree.**

3.     Introduction: It should be specified that the author refers to type I LTA.

**YL: I agree with both reviewers that type of LTA in *B. subtilis* should be specified.**

4.     Introduction: The first part of the introduction is very simplified, for example no lipid A is mentioned, nor the modification it can suffer to make it less anionic. I suggest rewriting and improving this paragraph.

**YL: I will expand the introductory part. For instance, lipid A and lipopolysaccharide will be mentioned.**

5.     Figure 1 and 5: Double bonds should look identical in the figures.

**YL: I will redraw the molecular figures to make the two lines in double bonds in the same thickness and redraw the glucosyl rings in chair conformation.**

6.     Strictly speaking the authors don’t show that it is D-alanine in the detected structures.

**YL: I will revise the statements concerning D-alanylation. I will briefly report that the lipid lysate has much more D-alanine than L-alanine.**

7.     Results/Discussion: What does the author think is the explanation that the new species only can be detected after a cold extraction?

**YL: It is possible that it is not stable at room temperature. I think the readers can explain this.**

8.     Methods: Why do the authors add sodium acetate to the growth medium before centrifugation of the culture?

**YL: It is out of respect to previous publications that teichoic acid-linked D-alanine tend to be most stable under mildly acidic condition. It is also part of my effort to standardize protocols. As the *B. subtilis* grow dense, pH typically gradually raises from neutrality to ~8.5. Unlike lipid extraction under low temperature, adding sodium acetate before centrifugation was not a required procedure as unbuffered cells also produced similar amount of the reported lipid species.**

9.     Page 2, left column, middle paragraph: I think it should say “adenylylation” not “adenylation”, because it is an AMP-transfer.

**YL: Strictly speaking “adenylylation” is correct. It will be corrected.**

10.   Page 2, left column, middle paragraph: Instead of “The functionally uncharacterized DltB appears to be an integral membrane protein…”, it would be better to write “is predicted to be…”.

**YL: I agree.**

11.   Page 2, left column, middle paragraph: It should say “O-acyltransferase”, not “o-acyltransferase”.

**YL: Thanks for pointing out.**

12.   In the legends to figures 3 and 4, it should say “acid” not “aicd”.

**YL: I was typing too fast.**

13.   Methods/Results: Why are the electronvolts sometimes “+” and sometimes “-“.

**YL: The sign of voltage values appears to follow the positive/negative mode of ionization. I had to specify the sign during the experiment.**

14.   Tables 2 and 4: The abbreviation FA for fatty acid should be explained.

**YL: I will add this abbreviation.**

15.   Page 8, left column, middle paragraph: The word “smaller” should be deleted.

**YL: I was emphasizing the relative ease of analyzing the smaller 1017 amu species than the larger 1045 amu species. As the sentence starts a paragraph, I agree it is better to remove “smaller”.**

16.   I agree with reviewer one that P is probably a better abbreviation of the phosphate group. 

**YL: I was trying to use 3-letter codes consistently. I will simplify it as “P”.**

# Peer review: Referee response for version 1

**DOI:** 10.5256/f1000research.8616.r12352

**Published:** 2016-02-25

**Authors:** Katarzyna Duda

**Affiliations:** 1 Junior Group of Allergobiochemistry, Research Center Borstel, Leibniz-Center for Medicine and Biosciences, Airway Research Center North (ARCN), Borstel, Germany

The work of Luo Y. describes an identification of the biosynthetic intermediates of the lipoteichoic acid utilizing mass spectrometrical approach.

Generally, the conclusions are based on the solid raw data, the title is appropriate, and the abstract provides subsequent summary of the article.

I have a few comments, which addressing can help to improve the manuscript:

1. In the abstract and introduction given description of LTA refers mainly to Type 1 of LTA. I would specify it, and in the introduction mention other types of LTA, that are structurally different (reviewed: Schneewind O., Missiakas D. J. Bacteriol. 2014, 196(6):1133.)
2. D-Alanylation of LTA 1 and 4 is indeed very common, for the reader of the paper would it be also of interest, to mention other LTA modifications.
3. The use of the sentence with the characterization of D- but not L-Alanine, is confusing, as to date all bacterial Ala is D-configured.
4. Fig. 1 - I suggest to draw sugars in chair conformation, and the double bonds of P- or COO- groups should be equally thick.
5. The given formula of glycerol is wrong - no double bond is present, better to use standard abreviation Gro, e.g. Gro-Glc-Glc (concerns also Table 1).
6. Phosphate groups are commonly abbreviated as P, not Pho. Table 1 - I would write LTA primer, not only LTA, otherwise confusing.
7. Page 4 . when describing peaks at 887 and 915 instead of using 30:0 and 32:0, please use 15:0+15:0, and 15:0+17:0, otherwise confusing.
8. Peak at 887 corresponds to 2x Hex, Gro, 2x15:0, Na, so corresponds to sodiated (but not dehydrated - this word should be removed) linker (Figure 2A). The confusion with dehydrated or not was present a few times in the text (e.g. 753 amu represents loss of glucose and not dehydrated glucose).
9. On page 4 - the QTRAP spectra are shown in Fig 3 - Fig 4, not as in text Fig 2 - Fig 4

I have read this submission. I believe that I have an appropriate level of expertise to confirm that it is of an acceptable scientific standard.

# Response

**Published:** 2016-03-03

**Authors:** Yu Luo

**Affiliations:** Department of Biochemistry, University of Saskatchewan, Canada

**I appreciate Dr. Duda’s time and comments. I will incorporate changes as described below in my response to Dr. Katarzyna Duda’s suggestions.**

1.      In the abstract and introduction given description of LTA refers mainly to Type 1 of LTA. I would specify it, and in the introduction mention other types of LTA, that are structurally different (reviewed: Schneewind O., Missiakas D. J. Bacteriol. 2014, 196(6):1133.)

**YL: I will mention the review and specify that *B. subtilis* produces Type 1 lipoteichoic acid.**

****

2.     D-Alanylation of LTA 1 and 4 is indeed very common, for the reader of the paper would it be also of interest, to mention other LTA modifications.

**YL: I will mention other types of lipoteichoic acid and that Type 1 and 4 lipoteichoic acids are commonly modified by D-alanine.**

****

3.     The use of the sentence with the characterization of D- but not L-Alanine, is confusing, as to date all bacterial Ala is D-configured.

**YL: It is actually necessary to specify D-alanine. MprF in *B. subtilis* is known to catalyze the synthesis of L-alanyl-phosphatidylglycerol form L-alanyl-tRNA and phosphatisylglycerol.**

****

4.     Fig. 1 - I suggest to draw sugars in chair conformation, and the double bonds of P- or COO- groups should be equally thick.

**YL: I will re-draw the structures with sugars in chair conformation, and revert to normal thickness in ChemDraw to make the double bonds equally thick.**

****

5.     The given formula of glycerol is wrong - no double bond is present, better to use standard abbreviation Gro, e.g. Gro-Glc-Glc (concerns also Table 1).

**YL: The 405 amu species likely represents the structure shown with a double bond in Table-1. It was a common fragment for this group of sodiated cations of DAG-Glc-Glc, indicating both fatty acyl tails are lost. The 405 amu fragment corresponds to the neutral loss of both fatty acyl free radicals, or equivalently a ketene and a peroxyfatty acid. The double bond formation in the glycerol residue is a consequence of such neutral losses.**

6.     Phosphate groups are commonly abbreviated as P, not Pho. Table 1 - I would write LTA primer, not only LTA, otherwise confusing.

**YL: I will rename Pho as P, and abbreviate LTA primer as LTAp.**

7.     Page 4 . when describing peaks at 887 and 915 instead of using 30:0 and 32:0, please use 15:0+15:0, and 15:0+17:0, otherwise confusing.

**YL: The (30:0) species was a mixture of (15:0-15:0), (14:0-16:0) and other diglucosyl-diacylglycerol. I will state that (15:0-15:0) is the most abundant form in the mixture.**

8.     Peak at 887 corresponds to 2x Hex, Gro, 2x15:0, Na, so corresponds to sodiated (but not dehydrated - this word should be removed) linker (Figure 2A). The confusion with dehydrated or not was present a few times in the text (e.g. 753 amu represents loss of glucose and not dehydrated glucose).

**YL: Ester and glycosidic bonds, as well as peptide bonds, are synthesized with the net effect of dehydration. Dissociation of the molecular ion is not equivalent to the hydrolysis process in solution where a water molecule is added back to the broken parts. One or the other fragment of the molecular ion has to bare the consequence of dehydration incurred during the biosynthesis process. For instance, the 753 amu fragment indeed represents the loss of a dehydrated glucose (162 amu) from the molecular ion of 915 amu. A neutral loss of 180 amu glucose would produce a fragment of 735 amu. It is true that the head group is not dehydrated in the lipid. However,** **the scan was indeed for precursor ions of the 347 amu dehydrated (-18 amu) and sodiated (+23 amu) diglucose (342 amu) fragment ion (342 – 18 + 23 = 347).**

9.     On page 4 - the QTRAP spectra are shown in Fig 3 - Fig 4, not as in text Fig 2 - Fig 4

**YL: They are indeed shown in Figures 2, 3 and 4.**
